# Conduction system pacing is superior to biventricular pacing in patients with heart failure: Insights from the pooled clinical studies

**DOI:** 10.3389/fphys.2023.1125340

**Published:** 2023-05-05

**Authors:** Jie Zhang, Feng Li, Zhi-Yuan Zhang, Fan Yang, Qi Kong, Jia-Yi Chen, Lei Zhang, Huan-Huan Liu, Xu-Fei Chen, Yu-Heng Ye, Ru-Xing Wang

**Affiliations:** Department of Cardiology, Wuxi People’s Hospital Affiliated to Nanjing Medical University, Wuxi, China

**Keywords:** left bundle branch area pacing, cardiac resynchronization therapy, biventricular pacing, heart failure, his bundle pacing (HBP)

## Abstract

**Background:** The effects of conduction system pacing (CSP) compared with conventional biventricular pacing (BVP) on heart function in patients with heart failure remain elusive.

**Methods:** PubMed, Embase, Cochrane’s Library and Web of science databases were searched up to 1 October 2022 for pertinent controlled studies. Random or fixed-effect model were used to synthesize the clinical outcomes. Subgroup analysis was performed to screen the potential confounding factors.

**Results:** Fifteen studies including 1,347 patients were enrolled. Compared with BVP, CSP was significantly associated with shortened QRS duration [WMD -22.51 ms; *p* = 0.000], improved left ventricular ejection fraction [WMD 5.53%; *p* = 0.000], improved NYHA grade [WMD -0.42; *p* = 0.000], higher response rate and lower heart failure rehospitalization rate. CSP resulted in better clinical outcomes in higher male proportion group than lower one compared with BVP. No significant differences of clinical outcomes were observed between left bundle branch area pacing (LBBaP) and his bundle pacing (HBP) except the pacing threshold. The pacing threshold of LBBaP was significantly lower than those in BVP and HBP.

**Conclusion:** This study suggests that CSP might be superior to conventional BVP for HF patients. In a higher male proportion group, CSP may be associated with more benefits than BVP.

**Systematic Review Registration:** https://www.crd.york.ac.uk/prospero/display_record.php?ID=CRD42022355991; Identifier: CRD42022355991.

## 1 Introduction

Cardiac resynchronization therapy (CRT) through biventricular pacing (BVP) has been proved to bring out clinical benefits in heart failure (HF) patients with reduced left ventricular ejection fraction (LVEF) and left bundle branch block (LBBB). However, CRT based on BVP was realized through non-physiological fusion of paced wavefronts from the right ventricular (RV) endocardium and left ventricular (LV) epicardium (Ploux et al., 2015). As a result, the super-response rates of BVP was relatively low (only 20%–30%) and a considerable number of patients (30% at most) may not derive clinical benefits from BVP (Ellenbogen and Huizar, 2012), which means we need more effective pacing strategies to deliver CRT. Recently, conduction system pacing (CSP), mainly including his bundle pacing (HBP) and left bundle branch area pacing (LBBaP) has emerged as a promising alternative CRT.

HBP has been established as a feasible pacing strategy to improve cardiac function in several researches and it can provide comparable LVEF improvement to BVP (Upadhyay et al., 2019a). However, the disadvantage of HBP lies in its high and unstable LBBB correction threshold. LBBaP was a novel technique developed by Huang et al. (2017). A series of case reports and observational studies demonstrated the feasibility and safety of LBBaP in HF patients meeting the criteria of CRT (Zhang et al., 2019; Guo et al., 2020; Wu et al., 2021). However, few studies compared the effectiveness between CSP and BVP. The purpose of this study is to determine whether there are differences in clinical prognosis and pacing parameters between CSP and BVP in HF patients who required CRT.

## 2 Methods

### 2.1 Study design

This meta-analysis was performed in accordance with the PRISMA guidelines. We conducted the meta-analysis registration on the PROSPERO platform (CRD42022355991).

### 2.2 Search strategy

A total of four databases (PubMed, Web of Science, Embase and the Cochrane Library) were systematically searched by two independent investigators (J. Zhang and F. Li) up to 1 October 2022. Search keywords included “conduction system pacing”, “His bundle pacing”, “left bundle area pacing”, “left bundle branch pacing” and “biventricular pacing”, and “cardiac resynchronization therapy”. We performed the search by using the keywords alone and following query formula” (conduction system pacing or His bundle pacing or left bundle branch pacing or left bundle branch area pacing) and (Biventricular pacing or cardiac resynchronization therapy)”. Studies reporting comparing outcomes between CSP and BVP were included. We also screened and conducted a manual search of the references of the original and review articles for potential studies not identified before.

### 2.3 Study selection

The titles, abstracts, and full texts were reviewed by two independent reviewers (J. Zhang and Z-Y Zhang) to select the eligible studies. The inclusion criteria are as follows: 1) randomized controlled trials, retrospective studies or prospective/observational studies. 2) studies comparing pacing outcomes between CSP and BVP in HF patients. 3) studies reporting on pacing outcomes during follow-up, including final QRS duration (QRSd), reduction in QRSd, final LVEF, improvement in LVEF, New York Heart Association (NYHA) grade, reduction in NYHA grade, echocardiographic, clinical CRT response rates and CRT super response rates. According to the references, echocardiographic CRT response was defined as at least 5% improvement of LVEF during follow-up (Guo et al., 2020). Clinical CRT response was defined as decreasing NYHA functional class for at least one grade at the last follow-up. CRT super response rate was defined as a significant improvement in LVEF for at least 20% or final LVEF≥50% (Wu et al., 2021). Review articles, letters, studies without original data, editorials, case reports, animal studies and protocols were excluded.

### 2.4 Data extraction and quality assessment

The data for eligible studies were extracted by two independent researchers (J. Zhang and F. Li) and any disagreements were resolved by a third researcher (R.-X. Wang). The extracted data mainly included study characteristics (such as first author, country, study design, publication year, sample size and follow-up time), patients’ demographic and clinical characteristics.

We use two appraisal tools to assess the study quality. The Cochrane’s Risk of Bias Tool Quality was used for evaluating the randomized controlled studies, and the Newcastle-Ottawa Scale (NOS) was used to assess the quality of non-randomized controlled studies.

### 2.5 Statistical analysis

We used the weighted mean difference (WMD) for continuous variables and risk ratio (RR) for categorical variables. The 95% confidence intervals (CI) for WMD and RR were calculated. The Stata (Version 16.0) was used for statistical analyses, and *p* < 0.05 was statistically significant.

The Chi-squared test and I-squared (I^2^) were used to assess the heterogeneity among studies. If the I^2^ value was less than 50% and/or *p* > 0.05 with the Chi-squared test, the between-study heterogeneity is not substantial, and a fixed-effect model was used. Otherwise, we used a random-effect model. Potential publication bias was assessed by the Egger regression asymmetry test. A sensitivity analysis with sequentially omitting one study method was conducted to evaluate the influence of a single study on the overall risk.

A subgroup analysis was also performed according to our previous reported methods (Li et al., 2022). A total of five confounding factors were screened, including study design (multi-center and single-center), CSP sample size (>20 and ≤20), male proportion (>50% and ≤50%), CSP types (LBBaP and HBP), and follow-up (≥12 months and <12 months).

## 3 Results

### 3.1 Study selection and quality assessment

**FIGURE 1:**
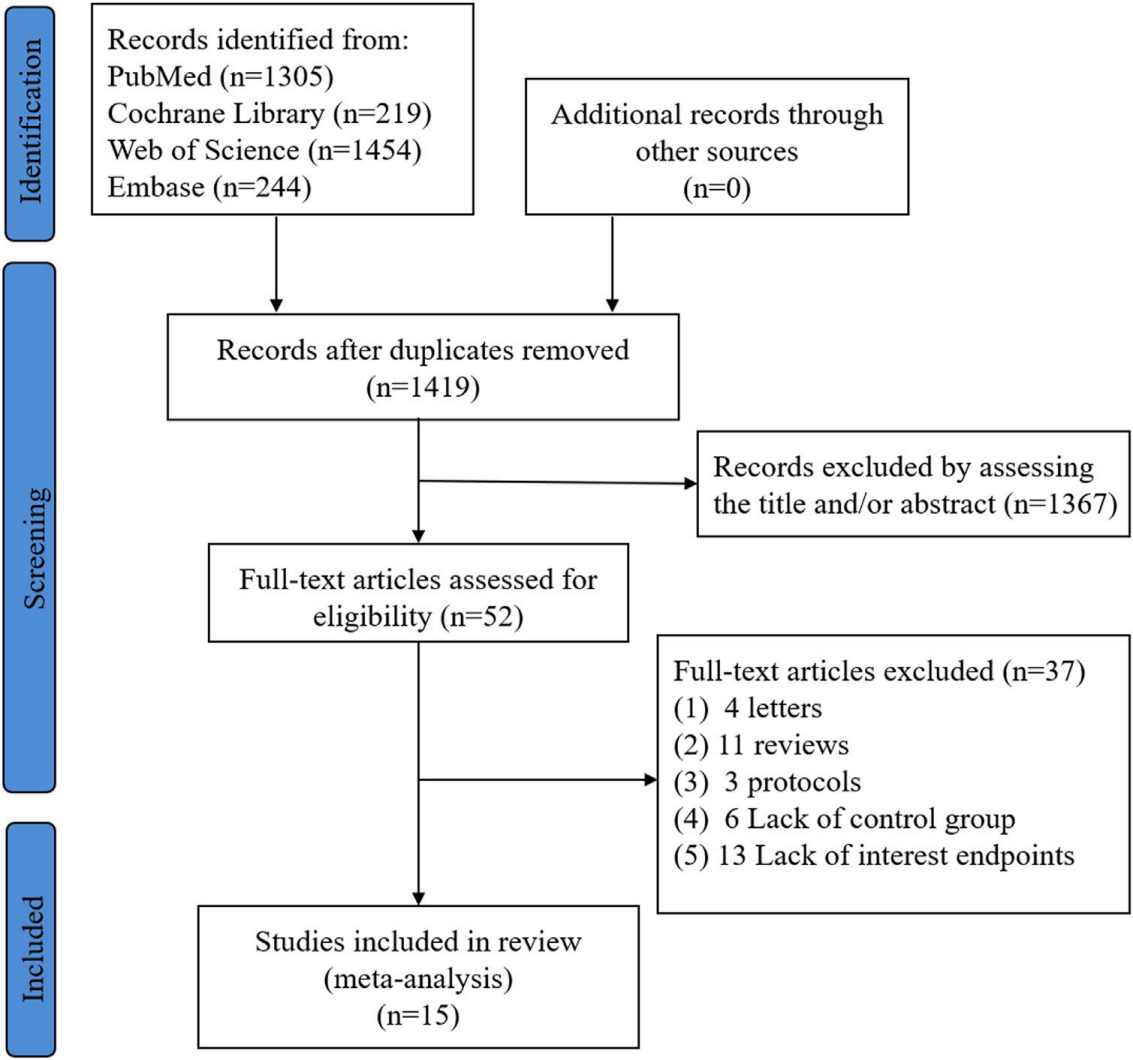
The flowchart of the study selection.

A total of fifteen studies including 1347 HF patients were eligible (Lustgarten et al., 2015; Upadhyay et al., 2019b; Vijayaraman et al., 2019; Guo et al., 2020; Li et al., 2020; Wang et al., 2020; Liu et al., 2021a; Vinther et al., 2021; Wu et al., 2021; Hua et al., 2022a; Chen et al., 2022; Moriña-Vázquez et al., 2022; Rademakers et al., 2022; Vijayaraman et al., 2022; Wang et al., 2022) the flowchart of study selection is displayed in Figure 1. The baseline characteristics of the eligible studies were presented in Table 1. Four of fifteen eligible studies were randomized controlled studies (Lustgarten et al., 2015; Upadhyay et al., 2019b; Vinther et al., 2021; Wang et al., 2022), and the literature quality was evaluated with the Cochrane’s Risk of Bias Tool (Supplementary Figure S1); meanwhile, the remaining eleven non-randomized studies (Vijayaraman et al., 2019; Guo et al., 2020; Li et al., 2020; Wang et al., 2020; Liu et al., 2021a; Wu et al., 2021; Hua et al., 2022a; Chen et al., 2022; Moriña-Vázquez et al., 2022; Rademakers et al., 2022; Vijayaraman et al., 2022) were evaluated with the Newcastle-Ottawa Scale (NOS) (Supplementary Table S1). The quality of all the studies were good.

**TABLE 1:** The baseline characteristics and procedure-related indexes of the eligible studies.

First author	Year	Study design	Country	CSP type	Sample size		Follow-up (months)	Male proportion (%)		Age (years)		Hypertension (%)		DM (%)	
					CSP group	BVP group		CSP group	BVP group	CSP group	BVP group	CSP group	BVP group	CSP group	BVP group
Wang	2022	Prospective randomized multi-center	China	LBBaP	20	20	6	35	65	62.3 ± 11.2	65.3 ± 10.6	NA	NA	NA	NA
Vijayaraman	2022	observational multi-center	America	LBBaP and HBP	258	219	27 ± 12	66	71	72 ± 13	72 ± 12	74	64	41	50
Chen	2022	prospective, observational multi-center	China	LBBaP	49	51	12	49.98	58.82	67.14 ± 8.88	64.37 ± 8.74	28.57	31.37	24.49	19.61
Moriña-Vázquez	2022	Retrospective single-center	Spain	HBP	52	51	12	63.4	68.6	64 (61–75)	68 (61–74)	75	74.5	28.8	39.2
Hua	2022	prospective, observational single-center	China	LBBaP	21	20	24	71.43	75	65.50 ± 6.91	67.50 ± 11.69	28.57	55.00	33.33	25.00
Rademakers	2022	prospective, single-center	Netherlands	LBBaP	40	40	6	48	68	68 ± 13	71 ± 9	85	80	20	23
Wu-1	2021	prospective, single-center	China	LBBaP	32	54	12	43.8	53.7	67.2 ± 13	68.3 ± 10	50	50	37.5	29.6
Wu-2	2021	prospective, single-center	China	HBP	49	54	12	63.3	53.7	68.3 ± 10	68.3 ± 10	40.8	50	12.2	29.6
Vinther	2021	prospective, randomized controlled single-center	Denmark	HBP	19	31	6	42	77	63.2 ± 9.2	67.4 ± 9.1	NA	NA	NA	NA
Liu	2021	prospective cohort muti-center	China	LBBaP	27	35	4.0 ± 1.4	51.9	57.1	72.9 ± 12.0	73.7 ± 14.6	40.7	45.7	33.3	22.9
Wang	2020	matched case-control single-center	China	LBBaP	10	30	6	90	76.7	64.80 ± 7.25	62.93 ± 10.33	NA	NA	NA	NA
Li	2020	prospective, observational multi-center	China	LBBaP	27	54	6	51.9	61.1	57.5 ± 9.8	58.5 ± 8.5	29.6	35.2	14.8	31.5
Guo	2020	prospective, observational single-center	China	LBBaP	21	21	6	42.9	42.9	66.1 ± 9.7	65.1 ± 7.5	42.9	33.3	38.1	4.8
Upadhyay	2019	prospective, randomized controlled multi-center	America	HBP	16	24	6	56.3	66.7	63.4 ± 13.3	65.5 ± 12.4	68.8	79.2	50	45.8
Vijayaraman	2019	retrospective, observational multi-center	America	HBP	27	27	14 ± 10	85	85	72 ± 15	72 ± 15	NA	NA	NA	NA
Lustgarten	2015	Crossover randomized controlled single-center	America	HBP	29	29	6	66	66	71.33	71.33	58.6	58.6	NA	NA

### 3.2 The final QRSd and shortening of QRSd

**FIGURE 2:**
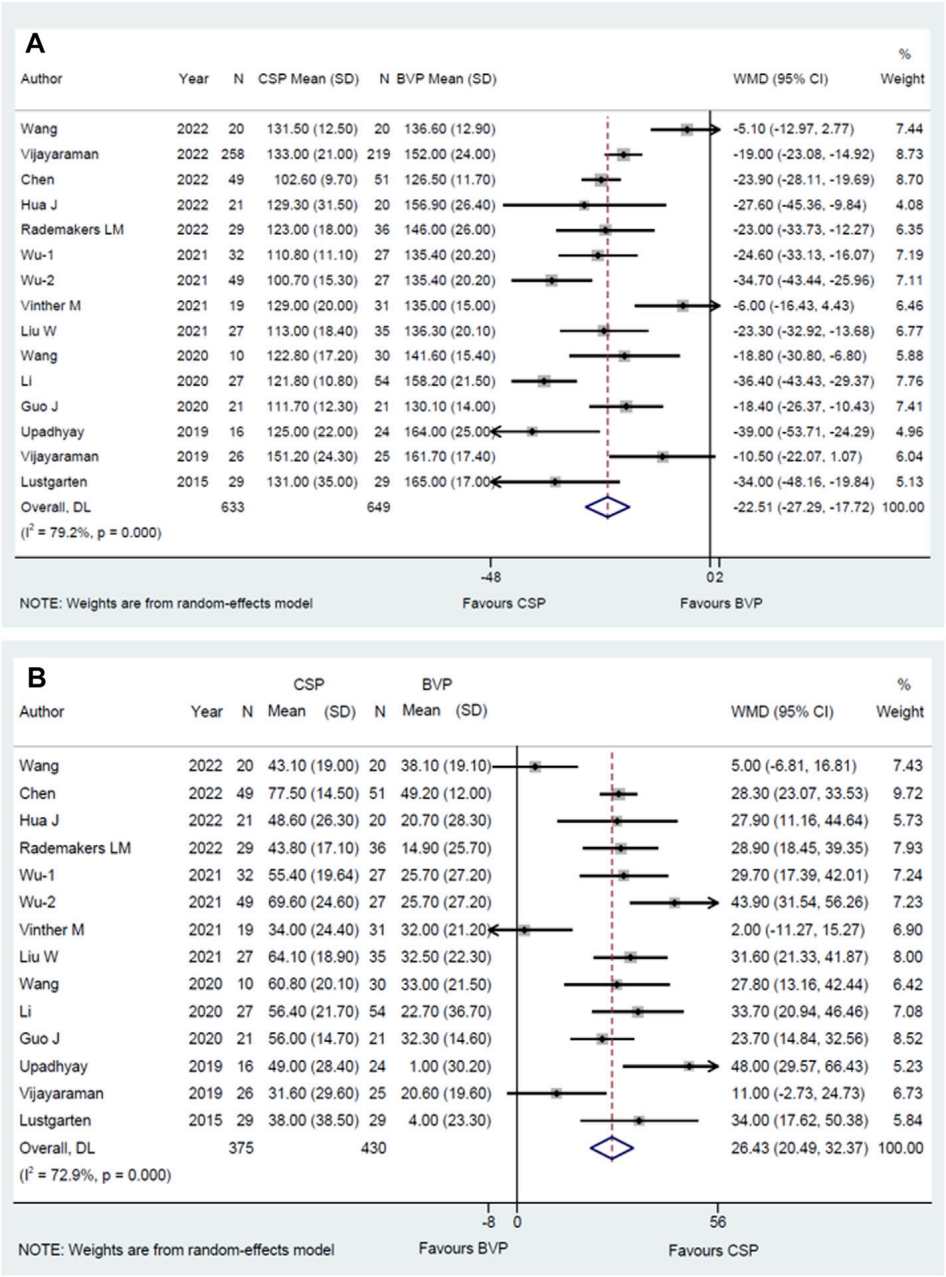
Forest plot of final QRSd and shortening of QRSd between CSP and BVP. **(A)** Final QRSd, **(B)** shortening of QRSd. CSP, conduction system pacing; BVP, biventricular pacing; WMD, weighted mean difference; CI, confidence interval.

All eligible studies (Lustgarten et al., 2015; Upadhyay et al., 2019b; Vijayaraman et al., 2019; Guo et al., 2020; Li et al., 2020; Wang et al., 2020; Liu et al., 2021a; Vinther et al., 2021; Wu et al., 2021; Hua et al., 2022a; Chen et al., 2022; Rademakers et al., 2022; Vijayaraman et al., 2022; Wang et al., 2022) including 1282 HF patients (633 patients for CSP, and 649 for BVP) reported the final QRSd, the average time of observation was 10.2 ± 7.2 months and thirteen studies (Lustgarten et al., 2015; Upadhyay et al., 2019b; Vijayaraman et al., 2019; Guo et al., 2020; Li et al., 2020; Wang et al., 2020; Liu et al., 2021a; Vinther et al., 2021; Wu et al., 2021; Hua et al., 2022a; Chen et al., 2022; Rademakers et al., 2022; Wang et al., 2022) reported the shortening of QRSd with a 8.8 ± 5.5 months time of observation. When compared with BVP in the last follow-up, CSP resulted in a narrower QRSd [WMD −22.51 ms; 95% CI (−27.29, −17.72); *p* = 0.000; I^2^ = 79.2%] (Figure 2A) and more shortening of QRSd [WMD 26.43 ms; 95% CI (20.49, 32.37); *p* = 0.000; I^2^ = 72.9%] (Figure 2B).

In addition, the significant treatment-covariate interaction was identified in the male proportion subgroup for more shortening of QRSd, including >50% subgroup [WMD 31.94 ms; 95% CI(24.40,39.47); *p* = 0.000; I^2^ = 56.6%] and ≤50% subgroup [WMD 20.34 ms; 95% CI (11.57,29.10); *p* = 0.000; I^2^ = 79.8%] with *p* = 0.049 for interaction (Supplementary Table S3). Similarly, the final QRSd of higher male proportion is narrower than the lower one, including >50% subgroup [WMD -26.72 ms; 95% CI (−33.40,-20.04); *p* = 0.000; I^2^ = 77.4%] and ≤50% subgroup [WMD −17.09 ms; 95% CI (−24.22,-9.95); *p* = 0.000; I^2^ = 80.3%], but it did not reach statistical significance with *p* = 0.053 for interaction (Supplementary Table S2). The results suggested that CSP tends to bring shorter QRSd in higher male proportion group when compared with BVP.

### 3.3 The final LVEF and the improvement of LVEF

**FIGURE 3:**
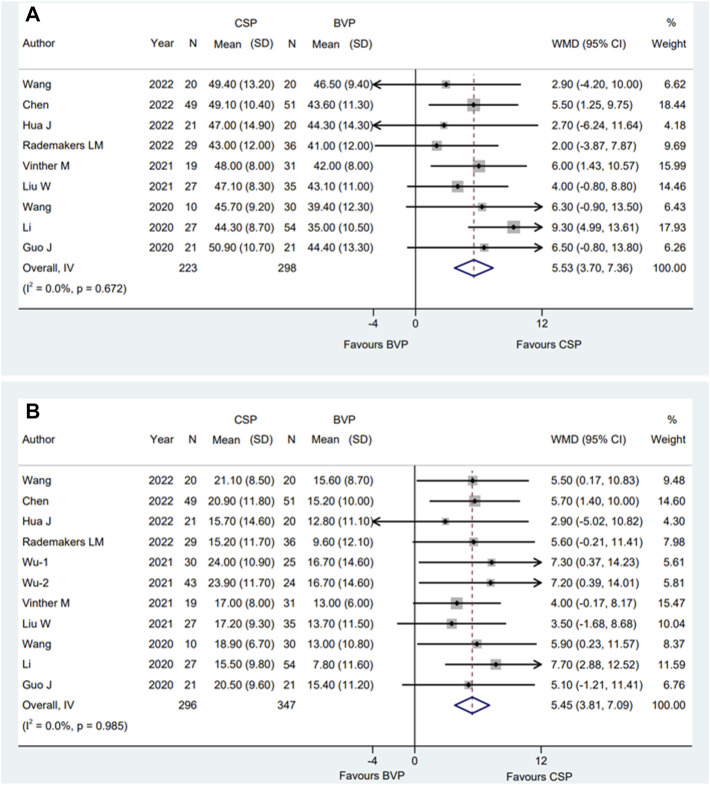
Forest plot of final LVEF and improvement of LVEF between CSP and BVP. **(A)** Final LVEF, **(B)** improvement of LVEF. CSP, conduction system pacing; BVP, biventricular pacing; WMD, weighted mean difference; CI, confidence interval.

The WMD and corresponding 95% CI of final LVEF was available from nine eligible studies (Guo et al., 2020; Li et al., 2020; Wang et al., 2020; Liu et al., 2021a; Vinther et al., 2021; Hua et al., 2022a; Chen et al., 2022; Rademakers et al., 2022; Wang et al., 2022), and the improvement of LVEF was also available from ten studies (Guo et al., 2020; Li et al., 2020; Wang et al., 2020; Liu et al., 2021a; Vinther et al., 2021; Wu et al., 2021; Hua et al., 2022a; Chen et al., 2022; Rademakers et al., 2022; Wang et al., 2022). The average time of observation was 8.4 ± 6.2 months for final LVEF, and 8.8 ± 6.0 months for the improvement of LVEF. When compared to BVP, CSP resulted in higher LVEF [WMD 5.53%; 95% CI (3.70, 7.36); *p* = 0.000; I^2^ = 0.0%] (Figure 3A) and a higher improvement in LVEF [WMD 5.45%; 95% CI (3.81,7.09); *p* = 0.000; I^2^ = 0.0%] (Figure 3B). The subgroup analysis for the final LVEF and the improvement of LVEF was shown in Supplementary Tables S4, 5.

### 3.4 The final NYHA and the improvement of NYHA

**FIGURE 4:**
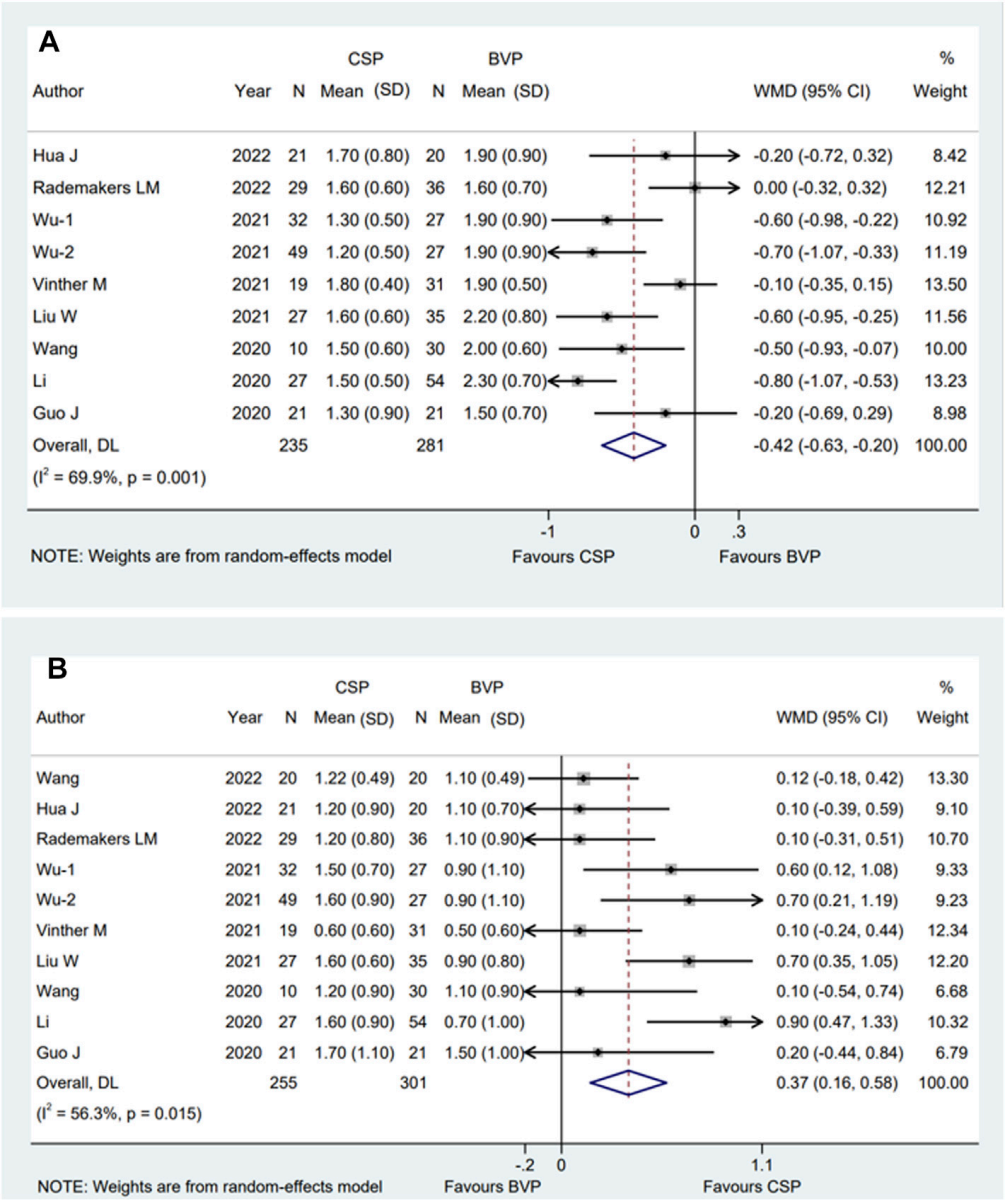
Forest plot of final NYHA grade and improvement of NYHA grade between CSP and BVP. **(A)** Final NYHA grade, **(B)** improvement of NYHA grade. CSP, conduction system pacing; BVP, biventricular pacing; WMD, weighted mean difference; CI, confidence interval.

All articles selected involving a total of eight eligible studies (Guo et al., 2020; Li et al., 2020; Wang et al., 2020; Liu et al., 2021a; Vinther et al., 2021; Wu et al., 2021; Hua et al., 2022a; Rademakers et al., 2022) who reported final NYHA and nine (Guo et al., 2020; Li et al., 2020; Wang et al., 2020; Liu et al., 2021a; Vinther et al., 2021; Wu et al., 2021; Hua et al., 2022a; Rademakers et al., 2022; Wang et al., 2022) reported the changes of NYHA. the average time of observation was 8.8 ± 6.6 months for final NYHA, and 8.4 ± 6.2 months for the improvement of NYHA. We used random-effect model to evaluate NYHA and the pooled results showed that compared with BVP, CSP was associated with significantly improved final NYHA grade [WMD −0.42; 95% CI (−0.63, −0.20); *p* = 0.000; I^2^ = 69.9%] (Figure 4A) and a higher change of NYHA [WMD 0.37; 95% CI (0.16, 0.58); *p* = 0.001; I^2^ = 56.3%] (Figure 4B).

The subgroup analysis showed that multi-center subgroup [WMD −0.73; 95% CI (−0.94, −0.52); *p* = 0.000; I^2^ = 0.0%] was significantly associated with improved NYHA class when compared with single-center group [WMD −0.32; 95% CI (−0.54, −0.10); *p* = 0.004; I^2^ = 56.9%] with *p* = 0.008 for interaction. The similar results also occurred in male proportion subgroup [>50% subgroup; WMD −0.63; 95% CI (−0.81, −0.45); *p* = 0.000; I^2^ = 14.6%, and ≤50% subgroup; WMD −0.20; 95% CI (−0.45, 0.05); *p* = 0.110; I^2^ = 52.4%, *p* = 0.007 for interaction]. Moreover, in the CSP sample size subgroup, the WMD was −0.26 [95% CI (−0.64, 0.12); *p* = 0.184; I^2^ = 59.7%] in the ≤20 group. In HBP group, the WMD was −0.38 [95% CI (−0.97, 0.20); *p* = 0.199; I^2^ = 85.7%] (Supplementary Table S6).

For the changes of NYHA, the subgroup analysis showed that CSP sample size >20 group [WMD 0.49; 95% CI (0.24,0.74); *p* = 0.000; I^2^ = 51.4%] was significantly associated with a higher improvement of NYHA class when compared with sample size ≤20 group [WMD 0.11; 95% CI (−0.10,0.32); *p* = 0.315; I^2^ = 0.0%] with *p* = 0.023 for interaction. Similar to what we observed in Final NYHA, the difference also shown in the male proportion subgroup [>50% group; WMD 0.54; 95% CI (0.24.0.85); *p* = 0.000; I^2^ = 53.4%, ≤50 group; WMD 0.18; 95% CI (0.01,0.36); *p* = 0.043; I^2^ = 0.0% *p* = 0.045 for interaction]. Moreover, in HBP group, the WMD was 0.37 [95% CI (−0.21,0.96); *p* = 0.210; I^2^ = 74.5%] (Supplementary Table S7).

### 3.5 Pacing thresholds, clinical response rate, echo response rate and super response rate

**FIGURE 5:**
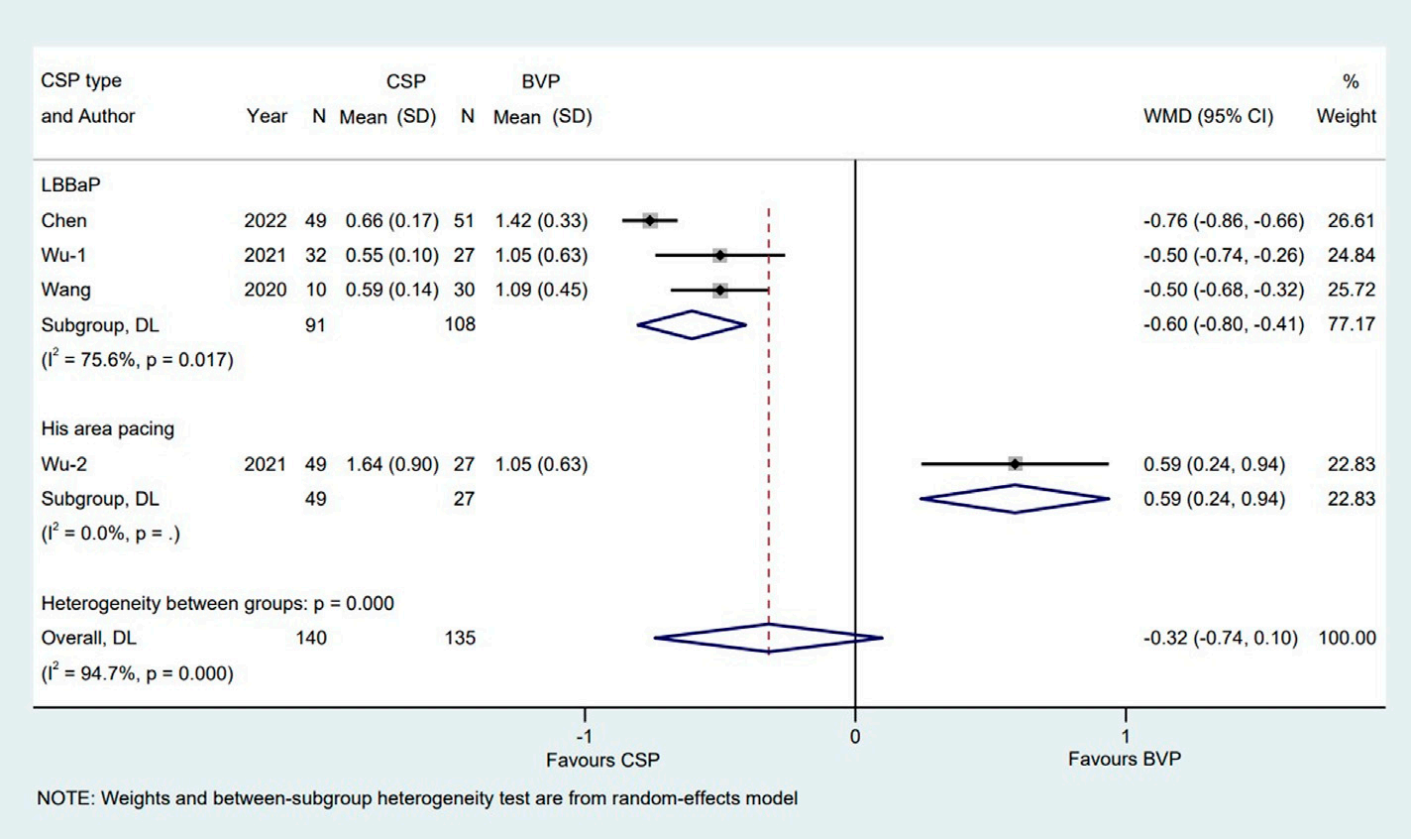
Forest plot of pacing thresholds between LBBaP, HBP and BVP LBBaP: left bundle branch area pacing; HBP, his bundle pacing; BVP, biventricular pacing; WMD, weighted mean difference; CI, confidence interval.

LV lead pacing threshold, one of the lead parameters, was used for analysis in BVP group. The pacing threshold was measured in different units, so we used the most frequently used unit V at 0.5 ms (V/0.5 ms) from three studies (Wang et al., 2020; Wu et al., 2021; Chen et al., 2022) for analysis, the average time of observation of the three studies was 10 ± 3.5 months. When compared to BVP group, LBBaP group provided a lower pacing threshold with a WMD of −0.60V/0.5 ms [95% CI (−0.80, −0.41); *p* = 0.000; I^2^ = 75.6%]. Conversely, compared with BVP group, HBP group was associated with a higher pacing threshold with a WMD of 0.59 V/0.5 ms [95% CI (0.24, 0.94); *p* = 0.001)] (Figure 5).

**FIGURE 6:**
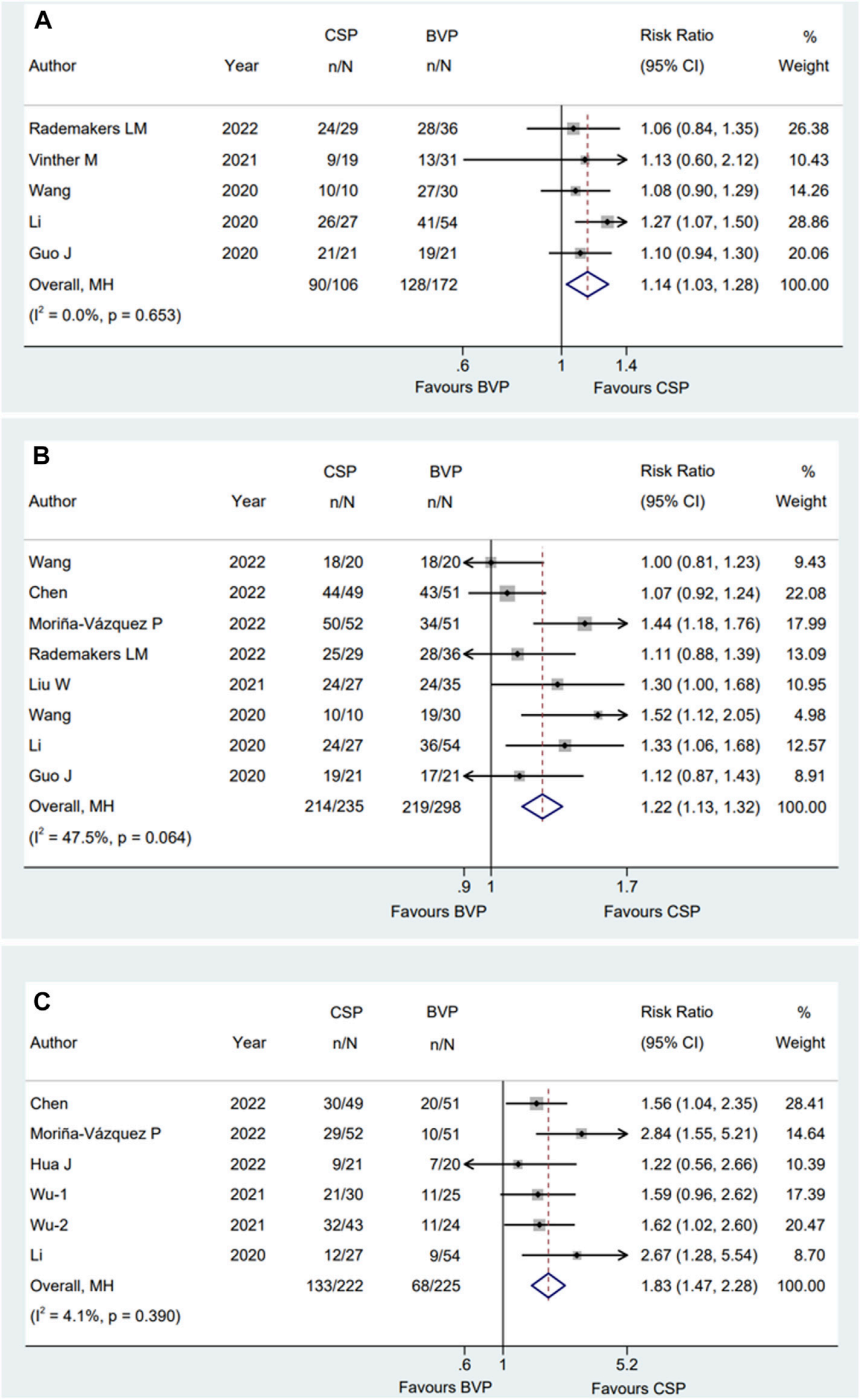
Forest plot of clinical response rate, echo response rate and super response rate between CSP and BVP. **(A)** Clinical response rate, **(B)** echo response rate, **(C)** super response rate. CSP, conduction system pacing; BVP, biventricular pacing; RR, risk ratio; CI, confidence interval.

**FIGURE 7:**
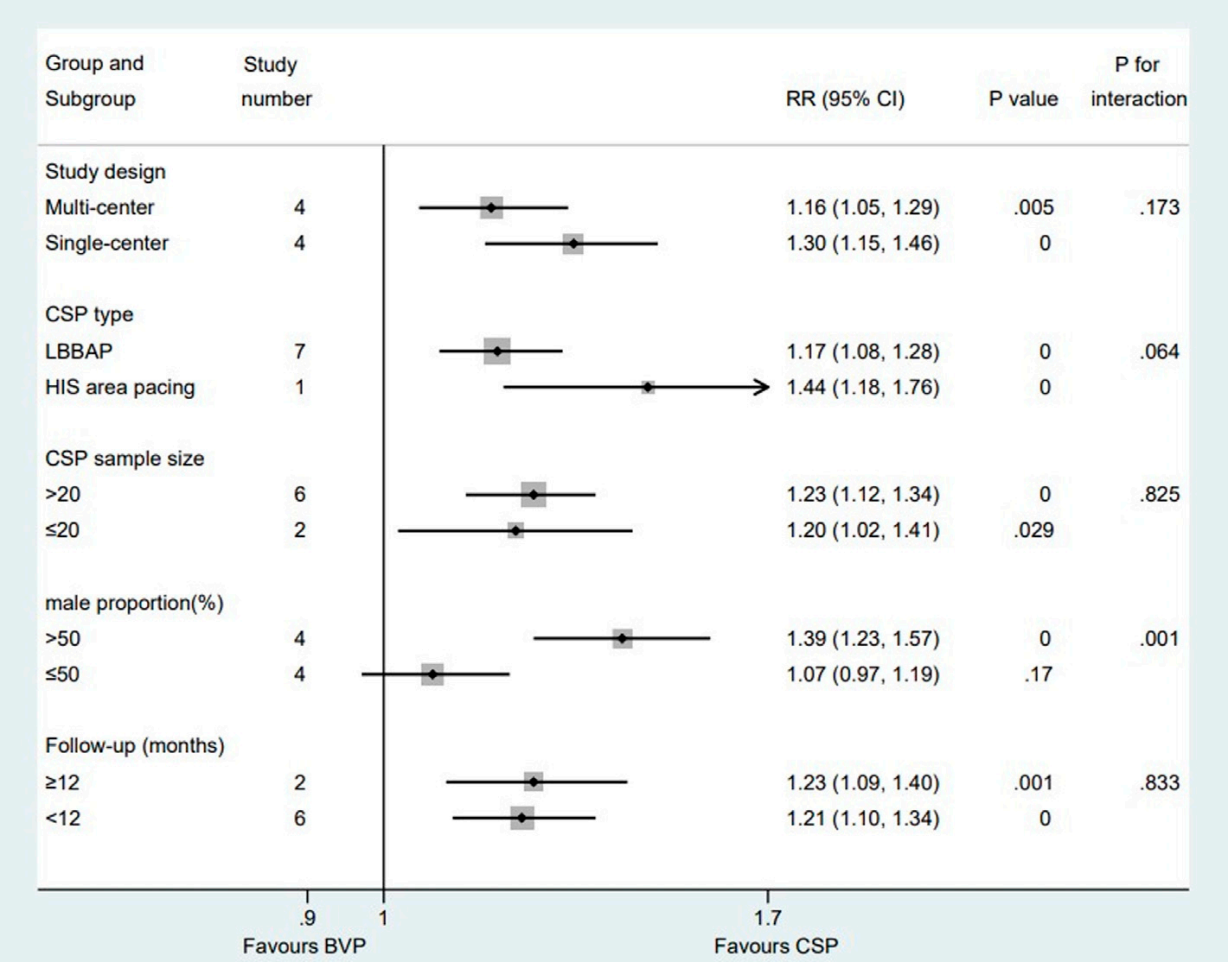
Subgroup analysis of echo response rate between CSP and BVP. Subgroup analysis was performed based on five confounding factors. CSP, conduction system pacing; BVP, biventricular pacing; RR, risk ratio; CI, confidence interval.

There is a total of five studies (Guo et al., 2020; Li et al., 2020; Wang et al., 2020; Vinther et al., 2021; Rademakers et al., 2022) reported clinical response rate, eight (Guo et al., 2020; Li et al., 2020; Wang et al., 2020; Liu et al., 2021a; Chen et al., 2022; Moriña-Vázquez et al., 2022; Rademakers et al., 2022; Wang et al., 2022) reported echo response rate and five (Li et al., 2020; Wu et al., 2021; Hua et al., 2022a; Chen et al., 2022; Moriña-Vázquez et al., 2022) for super response rate. The average time of observation was 6 months, 7.3 ± 3.0 months and 13.2 ± 6.6 months for clinical response rate, echo response rate and super response rate individually. Pooled results with fixed effect model showed that compared with patients who received BVP, patients who received CSP were more likely to achieve clinical CRT responses [RR:1.14; 95% CI (1.03,1.28); *p* = 0.014; I^2^ = 0.0%] (Figure 6A), echocardiographic CRT responses [RR:1.22; 95% CI (1.13,1.32); *p* = 0.000; I^2^ = 47.5%] (Figure 6B) and super CRT responses [RR:1.83; 95% CI (1.47,2.28); *p* = 0.000; I^2^ = 4.1%] (Figure 6C). There were no statistical differences between subgroups when it comes to clinical (Supplementary Table S8) and super CRT response rate (Supplementary Table S10). Subgroup analysis suggested that male proportion (%) > 50 group [RR:1.39; 95% CI (1.23,1.57); *p* = 0.000; I^2^ = 0.0%] had a higher echo CRT response rate than male proportion (%) ≤ 50 group [RR:1.07; 95% CI (0.97,1.19); *p* = 0.170; I^2^ = 0.0%] with *p* = 0.001 for interaction (Figure 7).

### 3.6 Publication bias and sensitivity analysis

Publication bias was not found from Egger’s test for all primary outcomes, sensitivity analysis of these outcomes was largely similar (Supplementary Table S11).

### 3.7 The rate of all-cause death, complication and HF rehospitalization

The data of all-cause death, complication and HF rehospitalization was presented in Supplementary Table S12. The average time of observation was 11.0 ± 7.1 months, 10.8 ± 7.4 months and 11.4 ± 7.6 months for all-cause death, complication and HF rehospitalization. The rate of all-cause death in CSP group (53/639 [8.3%]) is similar to that of BVP group (55/615 [8.9%]) [RR:0.81; 95% CI (0.58,1.14); *p* = 0.230]. The incidence of complications in CSP group (14/614 [2.3%]) is lower than that in BVP group (25/531 [4.7%]) but this did not reach statistical significance [RR:0.56; 95% CI (0.29,1.07); *p* = 0.079]. A total of 140 HFH (heart failure rehospitalization) occurred during the follow-up period. Pooled results with fixed effect model showed that there was a significant decrease in HFH in CSP group (51/571 [8.9%]) compared to that in BVP group (89/540 [16.5%]) [ RR:0.45; 95% CI (0.33,0.62); *p* = 0.000].

## 4 Discussion

In this meta-analysis, a total of fifteen eligible studies were enrolled to evaluate the clinical outcomes between two different pacing types (conduction system pacing vs biventricular pacing) for HF patients, and the main findings are as follows: 1) CSP is superior to conventional BVP for HF patients in terms of the clinical benefits, efficacy and prognosis; 2) CSP might be associated with more benefits than BVP In a higher male proportion group; 3) LBBaP may offer advantages over HBP for CRT due to a similar electromechanical resynchronization but lower pacing thresholds.

In order to further analyze the advantages of CSP, we conducted a subgroup analysis of five confounding factors. For the first time, we found that CSP had better efficacy (including shorter QRSd, higher echo response rate, and lower NYHA grade) with higher male proportion subgroup. Moreover, NYHA grade is lower in multi-centered groups than single-centered group and CSP sample size>20 groups were superior to that in ≤20 groups in terms of improvement of NYHA. Interestingly, CSP did not lead to lower NYHA grade and higher echo response rate than BVP (*p* > 0.05) in subgroups with lower male proportion, which is consistent with our findings that CSP tends to result in better cardiac function in male patients. In the CSP sample size ≤20 subgroup, CSP brings the final NYHA grade and improvement of NYHA similar to BVP, probably due to the inherent limitations of studies with small population included. Additionally, there was no statistical difference between HBP group and BVP group in final NYHA grade and improvement of NYHA, the reason for that might be that there are only two studies included and the population was too small to cause significant difference.

The controversies still remained on the efficacy of CRT for HF patients with different gender. The SMART-AV RCT showed a similar CRT response between male and female HF patients (Howell et al., 2021), while an Adapt Response RCT including 1,569 (43.3%) women patients with CRT indication found that the baseline characteristics and living quality between women and men were different, which may result in differences in clinical outcomes (Wilkoff et al., 2020). Waard *et al.* reported that women have significant reduced rates of death and HF hospitalization compared with men receiving CRT-D. what’s more, men were more likely to develop ventricular arrhythmias than women (de Waard et al., 2019). Whereas, our study showed that compared with BVP, male patients might contribute to better outcomes in CSP.

CSP mainly consisted of two different types, including HBP and LBBaP. Accumulated clinical studies revealed that patients with HF often have impaired His-Purkinje conduction, frequently manifested as LBBB. With the pacing lead directly implanted in the native conduction system, HBP can completely restore physiologic his-Purkinje conduction, which may be more beneficial to promote remodeling than BVP (Sharma et al., 2018; Upadhyay et al., 2019b; Huang et al., 2019). Arnold et al. (2018) found that compared with BVP, HBP provides greater improvement in hemodynamic parameters and better ventricular resynchronization, which further leads to the improvement of cardiac function. Therefore, His bundle pacing is considered to be a feasible alternative to conventional BVP in symptomatic HF patients. However, several limitations like high LBBB correction threshold and late threshold increase may restrict the wide clinical application of HBP (Hua et al., 2022b). Our meta-analysis included seven studies (Lustgarten et al., 2015; Upadhyay et al., 2019b; Vijayaraman et al., 2019; Vinther et al., 2021; Wu et al., 2021; Moriña-Vázquez et al., 2022; Vijayaraman et al., 2022) delivering HBP-CRT. The pacing thresholds were higher than BVP in five of them (Lustgarten et al., 2015; Upadhyay et al., 2019b; Vijayaraman et al., 2019; Vinther et al., 2021; Wu et al., 2021). Four studies found the improvement in LVEF was superior in patient who underwent HBP to those received BVP (Vinther et al., 2021; Wu et al., 2021; Moriña-Vázquez et al., 2022; Vijayaraman et al., 2022), while there was no difference between HBP and BVP in the His-SYNC pilot trail, it reported similar improvement in LVEF (7.9% vs 5.9%, *p* > 0.05), this may have been due to high crossover rate (48% of HBP group and 26% of BVP group) between the operation arms and the high proportion of patients with nonspecific intraventricular conduction defects (Upadhyay et al., 2019b). Similarly, HBP did not brought higher improvement than BVP in the study delivered by Lustgarten et al. probably due to this was a crossover design comparison study (Lustgarten et al., 2015), which gives us an inspiration to reduce the crossover rate between different CRT groups.

LBBaP is a new technique aimed at correcting the desynchrony of LBB conduction. It provides an alternative strategy for delivering CSP and can overcome many limitations of HBP. First of all, LBBaP corrects LBBB with a significantly lower pacing threshold than HBP, partly due to it delivers pacing beyond the site of conduction block. In addition, the lead is positioned closer to myocardial tissue, leading to higher R-wave amplitude with LBBaP. secondly, LBBaP has higher implant success rates, and the procedure time for LBBaP lead implantation was shorter than BVP. Thirdly, LBBaP can achieve left ventricular mechanical synchronization similar to that of HBP, but with better pacing parameters (Liu et al., 2021b; Wu et al., 2021; Moore et al., 2022). Recently, Palmisano et al. (2023) conducted a study comparing the long-term risk of device-related complications between CSP and BVP using propensity-matched analysis. The results showed that HBP showed a significantly higher risk of complications than LBBaP, which is another advantage for LBBaP. For the limitations above, HBP still showed some advantages over LBBaP, Ali et al. (2023) found that HBP delivered better ventricular resynchronization than LBBaP because right ventricular activation was slower during LBBaP. We observed that LBBaP had significantly narrower QRSd, more LVEF improvement, better NYHA class and higher CRT response rates than BVP in this meta-analysis. And there is no difference between LBBaP and HBP in clinical benefits and efficacy. Considering the benefits above, LBBaP appears to be a promising method for delivering CRT.

The VENUS trial (Lador et al., 2021) analyzed the primary outcome in two categories: High-volume centers (>20 patients enrolled) *versus* low-volume centers. Similarly, we divided the researches included into CSP sample size >20 and ≤20 group with reference to the VENUS trial. Our analysis found that in sample size ≤20 subgroup, CSP did not show statistical difference in the narrowing of QRSd and improvement of NYHA class, suggesting that when technical aspects of CSP are not mature, the effect of CSP might not be so significant.

The multicenter trial can enroll a larger number of subjects, cover a wide range of areas and avoid the limitations that may exist in single-center research, facilitating to a significant and credible the study conclusions. Our meta-analysis found that patients in multi-center groups were associated with significantly improved NYHA class compared with single-center group. The advantages of multi-center may account for the difference.

Moreover, the subgroup analysis found that no difference exists in both follow-up subgroups with all clinical outcomes. This may be attributed to the short follow-up period of the articles we included. Only two studies (Hua et al., 2022a; Vijayaraman et al., 2022) were followed up for about 24 months, and the remaining studies ranged from 6 to 12 months. One study found Permanent HBP was safe and effective during long-term follow-up with a median follow up of 3 years (Zanon et al., 2019). While another study revealed that the elevated capture thresholds, loss of His-bundle capture, and lead revision rates of HBP at intermediate follow-up (median 19.5 months) are of concern (Teigeler et al., 2021). The short-term and intermediate-term performance and safety of LBBaP has been proved, the comparison of long-term efficacy and safety between CSP and BVP remains unclear, studies recruiting more patients with longer follow-up periods and needed.

## 5 Limitation

Several potential limitations in our study should be highlighted. First, only four of fifteen eligible studies are RCT studies, and multiple potential confounding factors (such as selection bias and operator bias) might be existed despite of a comparable baseline characteristics between CSP and BVP group. Therefore, we conducted a subgroup analysis for different pacing outcomes between RCT subgroup and non-RCT subgroup, and the primary outcomes between two subgroups (such as the final QRSd, shortening of QRSd, the final LVEF, improvement of LVEF) showed the similar trends with our pooled results (Supplementary Table S13), indicated that our results are relatively robust. However, more randomized trials should be performed to further demonstrate our findings. Second, the number of patients included is relatively small, which means that the patients may not be sufficiently representative. Third, the follow-up was relatively not longer, the long-term (e.g., 3-year, 5-year or 10-year follow-up) effects of CSP on cardiac function and mechanical synchrony need to be confirmed by studies recruiting more patients with longer follow-up periods. Finally, since LBBaP was first developed by Huang’s team, a total eight of twelve eligible studies (Guo et al., 2020; Li et al., 2020; Wang et al., 2020; Liu et al., 2021a; Wu et al., 2021; Hua et al., 2022a; Chen et al., 2022; Wang et al., 2022) were derived from Chinese electrophysiology centers. Difference in proficiency and skills of the electrophysiologists may influence the comparative efficacy of LBBaP *versus* BVP for CRT.

## 6 Conclusion

This study suggests that CSP might be superior to conventional BVP for HF patients. In a higher male proportion group, CSP may be associated with more benefits than BVP.

## Data Availability

The original contributions presented in the study are included in the article/Supplementary Material, further inquiries can be directed to the corresponding author.
