# Supplementary material for: Conduction system pacing is superior to biventricular pacing in patients with heart failure: Insights from the pooled clinical studies
Source: Front Physiol. 2023 May 5;14:1125340. doi: 10.3389/fphys.2023.1125340 (PMC10196184; doi:10.3389/fphys.2023.1125340)
Supplement: Supplementary file 1 [file Table1.DOCX]

**Supplementary figure1** Quality evaluation of included RCTs

**
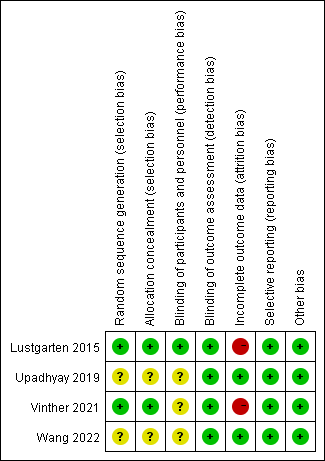
**

**Supplementary figure 1-A:** Risk of bias summary**.** The qualities of the included three RCTs, which were evaluated using the Cochrane bias risk assessment tool provided by the Review Manager. Different colors (green, yellow, and red) were used in the figure to indicate low-risk, unclear, and high-risk bias, respectively.


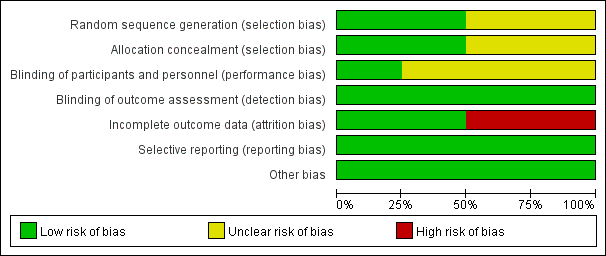


**Supplementary figure 1-B**：Risks of bias graph. The percentage of each level and level of each specific study item is presented. Abbreviations: RCT, randomized controlled trials.

**Supplementary table1** Quality assessment of eligible studies according to the Newcastle-Ottawa Quality Assessment Scale

| First author | Year | Selection | | | | Comparability | Outcome | | | Total stars |
| --- | --- | --- | --- | --- | --- | --- | --- | --- | --- | --- |
|  |  | Representative of the exposed cohort | Selection of the nonexposed cohort | Ascertainment of exposure | Demonstration that outcome of interest was not present at start of study | Comparability of cohorts on the basis  of the design or analysis | Assessment of outcome | Was follow-up long enough for outcomes to occur | Adequacy of follow-up of cohorts |  |
| Chen | 2022 | **☆** | **☆** | **☆** | **☆** | **☆** | **☆** | **☆** | **☆** | 8 |
| Vijayaraman | 2022 | **☆** | **☆** | **☆** | **☆** | **☆** | **☆** | **☆** | **☆** | 8 |
| Moriña-Vázquez | 2022 | **☆** | **☆** | **☆** | **☆** | **☆** | **☆** | **☆** | **☆** | 8 |
| Hua | 2022 | **☆** | **☆** | **☆** | **☆** | **☆** | **☆** | **☆** | **☆** | 8 |
| Rademakers | 2022 | **☆** | **☆** | **☆** | **☆** | **☆** | **☆** |  | **☆** | 7 |
| Wu-1 | 2021 | **☆** | **☆** | **☆** | **☆** | **☆** | **☆** | **☆** | **☆** | 8 |
| Wu-2 | 2021 | **☆** | **☆** | **☆** | **☆** | **☆** | **☆** | **☆** | **☆** | 8 |
| Liu | 2021 | **☆** | **☆** | **☆** | **☆** | **☆** | **☆** |  | **☆** | 7 |
| Wang | 2020 | **☆** | **☆** | **☆** | **☆** | **☆** | **☆** |  | **☆** | 7 |
| Li | 2020 | **☆** | **☆** | **☆** | **☆** | **☆** | **☆** |  | **☆** | 7 |
| Guo | 2020 | **☆** | **☆** | **☆** | **☆** | **☆** | **☆** |  | **☆** | 7 |
| Vijayaraman | 2019 | **☆** | **☆** | **☆** | **☆** | **☆** | **☆** | **☆** | **☆** | 8 |

**Supplementary table 2.** Subgroup analysis of the final Paced QRS duration between CSP group and BVP group

| **Subgroup Factors** | **Numbers of Study** | **WMD** | **95% CI** | **I^2^ (%)** | ***P* value** | ***P* for interaction** |
| --- | --- | --- | --- | --- | --- | --- |
| **study design** |  |  |  |  |  | 0.846 |
| Multi-centered | 7 | -22.04 | (-29.34,-14.74) | 86.8 | 0.000 |  |
| Single-centered | 8 | -23.01 | (-29.62,-16.41) | 67.5 | 0.000 |  |
| **CSP type** |  |  |  |  |  | 0.765 |
| LBBaP | 9 | -22.22 | (-28.17,-16.27) | 77.8 | 0.000 |  |
| HIS area pacing | 5 | -24.51 | (-38.28,-10.74) | 85.9 | 0.000 |  |
| **CSP sample size** |  |  |  |  |  | 0.239 |
| >20 | 11 | -24.77 | (-29.15,-20.40) | 69.4 | 0.000 |  |
| ≤20 | 4 | -16.25 | (-29.75,-2.75) | 83.7 | 0.018 |  |
| **Male proportion (%)** |  |  |  |  |  | 0.053 |
| >50 | 9 | -26.72 | (-33.40,-20.04) | 77.4 | 0.000 |  |
| ≤50 | 6 | -17.09 | (-24.22,-9.95) | 80.3 | 0.000 |  |
| **Follow-up** |  |  |  |  |  | 0.850 |
| ≥12 | 6 | -23.18 | (-28.46,-17.90) | 67.6 | 0.000 |  |
| <12 | 9 | -22.23 | (-30.62,-13.84) | 84.6 | 0.000 |  |

WMD: Weighted mean difference; CI: confidence interval; CSP: conduction system pacing; BVP: biventricular pacing; LBBaP: left bundle branch area pacing

**Supplementary table 3.** Subgroup analysis of the reduction of QRS duration between CSP group and BVP group

| **Subgroup Factors** | **Numbers of Study** | **WMD** | **95% CI** | **I^2^ (%)** | ***P* value** | ***P* for interaction** |
| --- | --- | --- | --- | --- | --- | --- |
| **study design** |  |  |  |  |  | 0.818 |
| Multi-centered | 6 | 25.63 | (15.58,35.67) | 80.4 | 0.000 |  |
| Single-centered | 8 | 27.13 | (19.12,35.15) | 68.7 | 0.000 |  |
| **CSP type** |  |  |  |  |  | 0.911 |
| LBBaP | 9 | 26.32 | (21.29,31.35) | 50.8 | 0.000 |  |
| HIS area pacing | 5 | 27.41 | (9.11,45.71) | 87.3 | 0.003 |  |
| **CSP sample size** |  |  |  |  |  | 0.357 |
| >20 | 10 | 29.07 | (24.64,33.50) | 39.1 | 0.000 |  |
| ≤20 | 4 | 19.83 | (0.67,38.99) | 86.2 | 0.043 |  |
| **Male proportion (%)** |  |  |  |  |  | 0.049 |
| >50 | 8 | 31.94 | (24.40,39.47) | 56.6 | 0.000 |  |
| ≤50 | 6 | 20.34 | (11.57,29.10) | 79.8 | 0.000 |  |
| **Follow-up** |  |  |  |  |  | 0.627 |
| ≥12 | 5 | 28.46 | (19.66,37.27) | 67.4 | 0.000 |  |
| <12 | 9 | 25.42 | (16.93,33.92) | 76.3 | 0.000 |  |

WMD: Weighted mean difference; CI: confidence interval; CSP: conduction system pacing; BVP: biventricular pacing; LBBaP: left bundle branch area pacing

**Supplementary table 4.** Subgroup analysis of final LVEF between CSP group and BVP group

| **Subgroup Factors** | **Numbers of Study** | **WMD** | **95% CI** | **I^2^ (%)** | ***P* value** | ***P* for interaction** |
| --- | --- | --- | --- | --- | --- | --- |
| **study design** |  |  |  |  |  | 0.551 |
| Multi-centered | 4 | 6.01 | （3.60,8.42） | 18.9 | 0.000 |  |
| Single-centered | 5 | 4.88 | （2.08,7.68） | 0.0 | 0.001 |  |
| **CSP type** |  |  |  |  |  | 0.826 |
| LBBaP | 8 | 5.44 | （3.45,7.43） | 0.0 | 0.000 |  |
| HIS area pacing | 1 | 6.00 | (1.43,10.57) | - | 0.010 |  |
| **CSP sample size** |  |  |  |  |  | 0.907 |
| >20 | 6 | 5.60 | (3.43,7.77) | 3.1 | 0.000 |  |
| ≤20 | 3 | 5.36 | (1.97,8.75) | 0.0 | 0.002 |  |
| **Male proportion (%)** |  |  |  |  |  | 0.403 |
| >50 | 4 | 6.43 | （3.64,9.21） | 10.5 | 0.000 |  |
| ≤50 | 5 | 4.85 | （2.43,7.27） | 0.0 | 0.000 |  |
| **Follow-up** |  |  |  |  |  | 0.751 |
| ≥12 | 2 | 4.98 | (1.14,8.82) | 0.0 | 0.011 |  |
| <12 | 7 | 5.69 | (3.61,7.77) | 0.0 | 0.000 |  |

WMD: Weighted mean difference; CI: confidence interval; CSP: conduction system pacing; BVP: biventricular pacing; LBBaP: left bundle branch area pacing

**Supplementary table 5.** Subgroup analysis of the improvement of LVEF between CSP group and BVP group

| **Subgroup Factors** | **Numbers of Study** | **WMD** | **95% CI** | **I^2^ (%)** | ***P* value** | ***P* for interaction** |
| --- | --- | --- | --- | --- | --- | --- |
| **study design** |  |  |  |  |  | 0.802 |
| Multi-centered | 4 | 5.68 | (3.25,8.11) | 0.0 | 0.000 |  |
| Single-centered | 7 | 5.26 | (3.03,7.49) | 0.0 | 0.000 |  |
| **CSP type** |  |  |  |  |  | 0.719 |
| LBBaP | 9 | 5.61 | (3.76,7.46) | 0.0 | 0.000 |  |
| HIS area pacing | 2 | 4.87 | (1.32,8.43) | 0.0 | 0.007 |  |
| **CSP sample size** |  |  |  |  |  | 0.642 |
| >20 | 8 | 5.73 | (3.72,7.74) | 0.0 | 0.000 |  |
| ≤20 | 3 | 4.90 | (2.06,7.75) | 0.0 | 0.001 |  |
| **Male proportion (%)** |  |  |  |  |  | 0.820 |
| >50 | 5 | 5.69 | (3.09,8.28) | 0.0 | 0.000 |  |
| ≤50 | 6 | 5.30 | (3.18,7.42 ) | 0.0 | 0.000 |  |
| **Follow-up** |  |  |  |  |  | 0.733 |
| ≥12 | 4 | 5.89 | (2.91,8.87) | 0.0 | 0.000 |  |
| <12 | 7 | 5.27 | (3.30,7.23) | 0.0 | 0.000 |  |

WMD: Weighted mean difference; CI: confidence interval; CSP: conduction system pacing; BVP: biventricular pacing ; LBBaP: left bundle branch area pacing

**Supplementary table 6.** Subgroup analysis of the final NYHA between CSP group and BVP group

| **Subgroup Factors** | **Numbers of Study** | **WMD** | **95% CI** | **I^2^ (%)** | ***P* value** | ***P* for interaction** |
| --- | --- | --- | --- | --- | --- | --- |
| **study design** |  |  |  |  |  | 0.008 |
| Multi-centered | 2 | -0.73 | (-0.94,-0.52) | 0.0 | 0.000 |  |
| Single-centered | 7 | -0.32 | (-0.54,-0.10) | 56.9 | 0.004 |  |
| **CSP type** |  |  |  |  |  | 0.882 |
| LBBaP | 7 | -0.43 | (-0.68,-0.19) | 66.0 | 0.001 |  |
| HIS area pacing | 2 | -0.38 | (-0.97,0.20) | 85.7 | 0.199 |  |
| **CSP sample size** |  |  |  |  |  | 0.387 |
| >20 | 7 | -0.46 | (-0.71,-0.21) | 68.3 | 0.000 |  |
| ≤20 | 2 | -0.26 | (-0.64,0.12) | 59.7 | 0.184 |  |
| **Male proportion (%)** |  |  |  |  |  | 0.007 |
| >50 | 5 | -0.63 | (-0.81,-0.45) | 14.6 | 0.000 |  |
| ≤50 | 4 | -0.20 | (-0.45,0.05) | 52.4 | 0.110 |  |
| **Follow-up** |  |  |  |  |  | 0.359 |
| ≥12 | 3 | -0.55 | (-0.81,-0.29) | 17.6 | 0.000 |  |
| <12 | 6 | -0.37 | (-0.66,-0.08) | 77.5 | 0.012 |  |

WMD: Weighted mean difference; CI: confidence interval; CSP: conduction system pacing; BVP: biventricular pacing ; LBBaP: left bundle branch area pacing

**Supplementary table 7.** Subgroup analysis of the changes in NYHA between CSP group and BVP group

| **Subgroup Factors** | **Numbers of Study** | **WMD** | **95% CI** | **I^2^ (%)** | ***P* value** | ***P* for interaction** |
| --- | --- | --- | --- | --- | --- | --- |
| **study design** |  |  |  |  |  | 0.260 |
| Multi-centered | 3 | 0.56 | (0.08,1.03) | 81.1 | 0.022 |  |
| Single-centered | 7 | 0.26 | (0.07,0.46) | 16.3 | 0.009 |  |
| **CSP type** |  |  |  |  |  | 0.988 |
| LBBaP | 8 | 0.37 | (0.13,0.61) | 57.5 | 0.003 |  |
| HIS area pacing | 2 | 0.37 | (-0.21,0.96) | 74.5 | 0.210 |  |
| **CSP sample size** |  |  |  |  |  | 0.023 |
| >20 | 7 | 0.49 | (0.24,0.74) | 51.4 | 0.000 |  |
| ≤20 | 3 | 0.11 | (-0.10,0.32) | 0.0 | 0.315 |  |
| **Male proportion (%)** |  |  |  |  |  | 0.045 |
| >50 | 5 | 0.54 | (0.24,0.85) | 53.4 | 0.000 |  |
| ≤50 | 5 | 0.18 | (0.01,0.36) | 0.0 | 0.043 |  |
| **Follow-up** |  |  |  |  |  | 0.534 |
| ≥12 | 3 | 0.47 | (0.11,0.83) | 39.6 | 0.011 |  |
| <12 | 7 | 0.33 | (0.07,0.59) | 63.5 | 0.014 |  |

WMD: Weighted mean difference; CI: confidence interval; CSP: conduction system pacing; BVP: biventricular pacing; LBBaP: left bundle branch area pacing

**Supplementary table 8.** Subgroup analysis of Clinical response rate between CSP group and BVP group

| **Subgroup Factors** | **Numbers of Study** | **Risk ratio** | **95% CI** | **I^2^ (%)** | ***P* value** | ***P* for interaction** |
| --- | --- | --- | --- | --- | --- | --- |
| **study design** |  |  |  |  |  | 0.182 |
| Multi-centered | 1 | 1.27 | (1.07,1.50) | - | 0.005 |  |
| Single-centered | 4 | 1.09 | (0.95,1.26) | 0.0 | 0.194 |  |
| **CSP type** |  |  |  |  |  | 0.963 |
| LBBaP | 4 | 1.15 | (1.04,1.26) | 0.0 | 0.005 |  |
| HIS area pacing | 1 | 1.13 | (0.60,2.12) | - | 0.704 |  |
| **CSP sample size** |  |  |  |  |  | 0.842 |
| >20 | 3 | 1.15 | (1.03,1.29) | 0.0 | 0.012 |  |
| ≤20 | 2 | 1.12 | (0.85,1.47) | 0.0 | 0.425 |  |
| **Male proportion (%)** |  |  |  |  |  | 0.302 |
| >50 | 2 | 1.22 | (1.06,1.37) | 50.5 | 0.001 |  |
| ≤50 | 3 | 1.09 | (0.92,1.29) | 0.0 | 0.313 |  |

RR: Risk Ratio; CI: confidence interval; CSP: conduction system pacing; BVP: biventricular pacing; LBBaP: left bundle branch area pacing

**Supplementary table 9.** Subgroup analysis of Echo response rate between CSP group and BVP group

| **Subgroup Factors** | **Numbers of Study** | **Risk ratio** | **95% CI** | **I^2^ (%)** | ***P* value** | ***P* for interaction** |
| --- | --- | --- | --- | --- | --- | --- |
| **study design** |  |  |  |  |  | 0.173 |
| Multi-centered | 4 | 1.16 | (1.05,1.29) | 43.6 | 0.005 |  |
| Single-centered | 4 | 1.30 | (1.15,1.46) | 43.6 | 0.000 |  |
| **CSP type** |  |  |  |  |  | 0.064 |
| LBBaP | 7 | 1.17 | (1.08,1.28) | 32.0 | 0.000 |  |
| HIS area pacing | 1 | 1.44 | (1.18,1.76) | - | 0.000 |  |
| **CSP sample size** |  |  |  |  |  | 0.825 |
| >20 | 6 | 1.23 | (1.12,1.34) | 35.7 | 0.000 |  |
| ≤20 | 2 | 1.20 | (1.02,1.41) | 81.3 | 0.029 |  |
| **Male proportion (%)** |  |  |  |  |  | 0.001 |
| >50 | 4 | 1.39 | (1.23,1.57) | 0.0 | 0.000 |  |
| ≤50 | 4 | 1.07 | (0.97,1.19) | 0.0 | 0.170 |  |
| **Follow-up** |  |  |  |  |  | 0.833 |
| ≥12 | 2 | 1.23 | (1.09,1.40) | 83.2 | 0.001 |  |
| <12 | 6 | 1.21 | (1.10,1.34) | 32.6 | 0.000 |  |

RR: Risk Ratio; CI: confidence interval; CSP: conduction system pacing; BVP: biventricular pacing; LBBaP: left bundle branch area pacing

**Supplementary table 10.** Subgroup analysis of super response rate between CSP group and BVP group

| **Subgroup Factors** | **Numbers of Study** | **Risk ratio** | **95% CI** | **I^2^ (%)** | ***P* value** | ***P* for interaction** |
| --- | --- | --- | --- | --- | --- | --- |
| **study design** |  |  |  |  |  | 0.977 |
| Multi-centered | 2 | 1.82 | (1.28,2.60) | 37.3 | 0.001 |  |
| Single-centered | 4 | 1.83 | (1.38,2.43) | 17.2 | 0.000 |  |
| **CSP type** |  |  |  |  |  | 0.295 |
| LBBaP | 4 | 1.66 | (1.27,2.18) | 0.0 | 0.000 |  |
| HIS area pacing | 2 | 2.13 | (1.46,3.11) | 53.8 | 0.000 |  |
| **Male proportion (%)** |  |  |  |  |  | 0.242 |
| >50 | 4 | 2.04 | (1.51,2.77) | 29.5 | 0.000 |  |
| ≤50 | 2 | 1.57 | (1.15,2.16) | 0.0 | 0.005 |  |
| **Follow-up** |  |  |  |  |  | 0.280 |
| ≥12 | 5 | 1.75 | (1.39,2.20) | 0.0 | 0.000 |  |
| <12 | 1 | 2.67 | (1.28,5.54) | - | 0.008 |  |

RR: Risk Ratio; CI: confidence interval; CSP: conduction system pacing; BVP: biventricular pacing; LBBaP: left bundle branch area pacing

**Supplementary table 11**. Publication bias and sensitivity analysis of primary outcomes.

| **Primary outcomes** | **Sensitivity analysis** | | ***P* for egger’s test** |
| --- | --- | --- | --- |
|  | Lower range | Upper range |  |
| Final QRSd | WMD=-23.86ms ,95%CI(-28.28,-19.43) | WMD=-21.25ms ,95%CI(-25.79,-16.71) | 0.763 |
| Shortening of QRSd | WMD=24.46ms ,95%CI(18.33, 30.59) | WMD= 27.78ms ,95%CI(22.10, 33.46) | 0.850 |
| Final LVEF | WMD=4.71% ,95%CI(2.69, 6.72) | WMD=5.91% ,95%CI(3.99, 7.83) | 0.231 |
| Improvement of LVEF | WMD=5.16% ,95%CI(3.41,6.90) | WMD=5.72% ,95%CI(3.94,7.50) | 0.765 |
| Final NYHA | WMD=-0.48 ,95%CI(-0.69,-0.27) | WMD=-0.36 ,95%CI(-0.56,-0.15) | 0.981 |
| Improvement of NYHA | WMD=0.31 ,95%CI(0.11,0.50) | WMD=0.40 ,95%CI(0.18,0.63) | 0.782 |
| Pacing thresholds | WMD=-0.60 ,95%CI(-0.80,-0.41) | WMD=-0.15 ,95%CI(-0.73,0.42) | 0.094 |
| Clinical response rate | RR=1.09, 95%CI(0.95,1.26) | RR=1.17, 95%CI(1.04,1.32) | 0.781 |
| Echo response rate | RR=1.20, 95%CI(1.11,1.30) | RR=1.27, 95%CI(1.16,1.39) | 0.176 |
| Super response rate | RR=1.75, 95%CI(1.39,2.20) | RR=1.93, 95%CI(1.49,2.51) | 0.478 |
| All-cause death rate | RR=0.77, 95%CI(0.55,1.09) | RR=2.25, 95%CI(0.59,8.49) | 0.095 |
| Complication rate | RR=0.45, 95%CI(0.21,0.96) | RR=0.70, 95%CI(0.36,1.37) | 0.070 |
| HF rehospitalization rate | RR=0.43, 95%CI(0.31,0.60) | RR=0.55, 95%CI(0.25,1.23) | 0.352 |

WMD: Weighted mean difference; CI: confidence interval;

**Supplementary table 12** Rate of all-cause death, complication and HF rehospitalization of eligible studies

| First author | Year | Sample size | | All-cause death rate (%) | | Complication rate (%) | | HF rehospitalization rate (%) | |
| --- | --- | --- | --- | --- | --- | --- | --- | --- | --- |
|  |  | **CSP group** | **BVP group** | **CSP group** | **BVP group** | **CSP group** | **BVP group** | **CSP group** | **BVP group** |
| Wang | 2022 | 20 | 20 | 0 | 0 | 1(5) | 1(5) | 0 | 0 |
| vijayaraman | 2022 | 258 | 219 | 45(17) | 52(24) | 5(1.9) | 6(2.8) | 38(15) | 74(34) |
| Chen | 2022 | 49 | 51 | 0 | 0 | 0 | 5(9.8) | 2(4.08) | 5(9.8) |
| Moriña-Vázquez | 2022 | 52 | 51 | 0 | 0 | 1(1.9) | 2(3.9) | NA | NA |
| Hua | 2022 | 21 | 20 | 1(4.8) | 1(5) | 0 | 0 | 4(19) | 4(20) |
| Rademakers | 2022 | 40 | 40 | 2(5) | 2(5) | 0 | 0 | 1(2.5) | 2(5) |
| Wu-1 | 2021 | 32 | 54 | 0 | 0 | 0 | 2(3.7) | 0 | 3(5.6) |
| Wu-2 | 2021 | 49 | 54 | 2(4.1) | 0 | 1(2) | 2(3.7) | 2(4.1) | 3(5.6) |
| Vinther | 2021 | 19 | 31 | 0 | 0 | 1 | 3 | 0 | 0 |
| Liu | 2021 | 27 | 35 | NA | NA | NA | NA | NA | NA |
| Wang | 2020 | 10 | 30 | 0 | 0 | 0 | NA | 0 | 1(3.3) |
| Li | 2020 | 27 | 54 | 0 | 0 | 0 | NA | 0 | 0 |
| Guo | 2020 | 21 | 21 | 0 | 0 | 4 | 3 | 0 | 0 |
| Upadhyay | 2019 | 16 | 24 | 2(12.5) | 0 | 1 | 3 | NA | NA |
| Vijayaraman | 2019 | 25 | 25 | 1（4） | NA | NA | NA | 4（16） | NA |
| Lustgarten | 2015 | 29 | 29 | NA | NA | NA | NA | NA | NA |
| Total results |  |  |  | 53(8.3) | 55(8.9) | 14(2.3) | 25(4.7) | 51(8.9) | 89(16.5) |
| RR with 95%CI |  |  |  | 0.81(0.58,1.14) | | 0.56(0.29,1.07) | | 0.45(0.33,0.62) | |
| *P* value |  |  |  | 0.230 | | 0.079 | | 0.000 | |

Note: Complications include implant-related complications( ie, pneumothorax, perforation, pericardial effusion, implant site hematoma, implant site infection), lead-related complications (ie, lead dislodgment, lead fracture, or inability to pace owing to high threshold or phrenic capture) and other complications like stroke, arrhythmias.

RR: Risk Ratio; CI: confidence interval; CSP: conduction system pacing; BVP: biventricular pacing;

**Supplementary table 13** Subgroup analysis of primary pacing outcomes between randomized studies and non-randomized studies

| **Pacing outcomes** | **Numbers of Study** | **WMD/RR** | **95% CI** | **I^2^ (%)** | ***P* value** | ***P* for interaction** |
| --- | --- | --- | --- | --- | --- | --- |
| **Final QRSd** |  |  |  |  |  | 0.675 |
| Randomized | 4 | -20.15 | (-36.80,-3.50) | 88.4 | 0.018 |  |
| Non-randomized | 10 | -23.83 | (-28.07,-19.59) | 68.0 | 0.000 |  |
| **Shortening of QRSd** |  |  |  |  |  | 0.497 |
| Randomized | 4 | 21.39 | (0.74,42.05) | 87.4 | 0.042 |  |
| Non-randomized | 10 | 28.70 | (24.37,33.03) | 37.4 | 0.000 |  |
| **Final LVEF** |  |  |  |  |  | 0.800 |
| Randomized | 2 | 5.09 | (1.25,8.93) | 0.0 | 0.009 |  |
| Non-randomized | 7 | 5.66 | (3.58,7.73) | 0.0 | 0.000 |  |
| **Improvement of LVEF** | |  |  |  |  | 0.543 |
| Randomized | 2 | 4.57 | (1.28,7.85) | 0.0 | 0.006 |  |
| Non-randomized | 7 | 5.73 | (3.85,7.64) | 0.0 | 0.000 |  |
| **Complication rate** |  |  |  |  |  | 0.897 |
| Randomized | 3 | 0.61 | (0.16,2.29) | 0.0 | 0.409 |  |
| Non-randomized | 5 | 0.55 | (0.26,1.14) | 0.0 | 0.109 |  |

WMD: Weighted mean difference; RR: Risk Ratio; CI: confidence interval;
